# Supplementary material for: Physics-based broadband characterization of weak earthquakes
Source: Nat Commun. 2025 Dec 8;16:11300. doi: 10.1038/s41467-025-66461-w (PMC12722209; doi:10.1038/s41467-025-66461-w)
Supplement: Supplementary file 1 — Supplementary Information [file 41467_2025_66461_MOESM1_ESM.pdf]

Supplementary Information: Physics-based broadband characterization of weak earthquakes

František Gallovič*[1], Sara Sgobba[2], and Ľubica Valentová K.[1]

[1]Department of Geophysics, Faculty of Mathematics and Physics, Charles University, Prague, Czech Republic
[2]Istituto Nazionale di Geofisica e Vulcanologia, Milan, Italy

*Frantisek.Gallovic@matfyz.cuni.cz

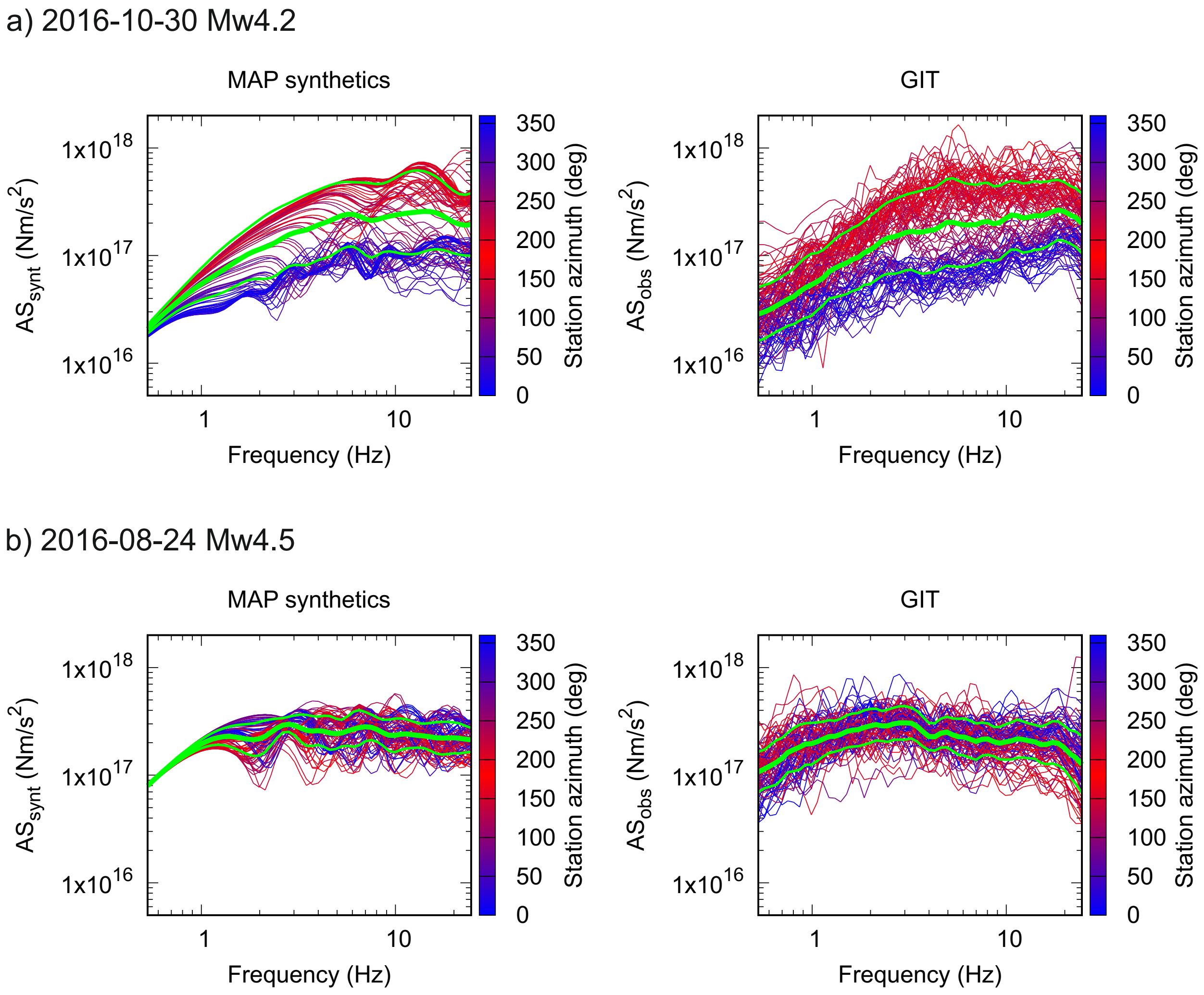

Figure S1: Comparison of (left) synthetic apparent spectra (AS) of the maximum a-posteriori (MAP) model and (right) empirical AS from the Generalized Inversion Technique (GIT) for a) the directive Mw4.2, and b) the non-directive Mw4.5 event. AS of individual stations are color-coded by station azimuth. Thick and thin green lines correspond to mean and one standard deviation across stations, respectively. These AS were used to calculate the spectral bias in Fig. 3a.

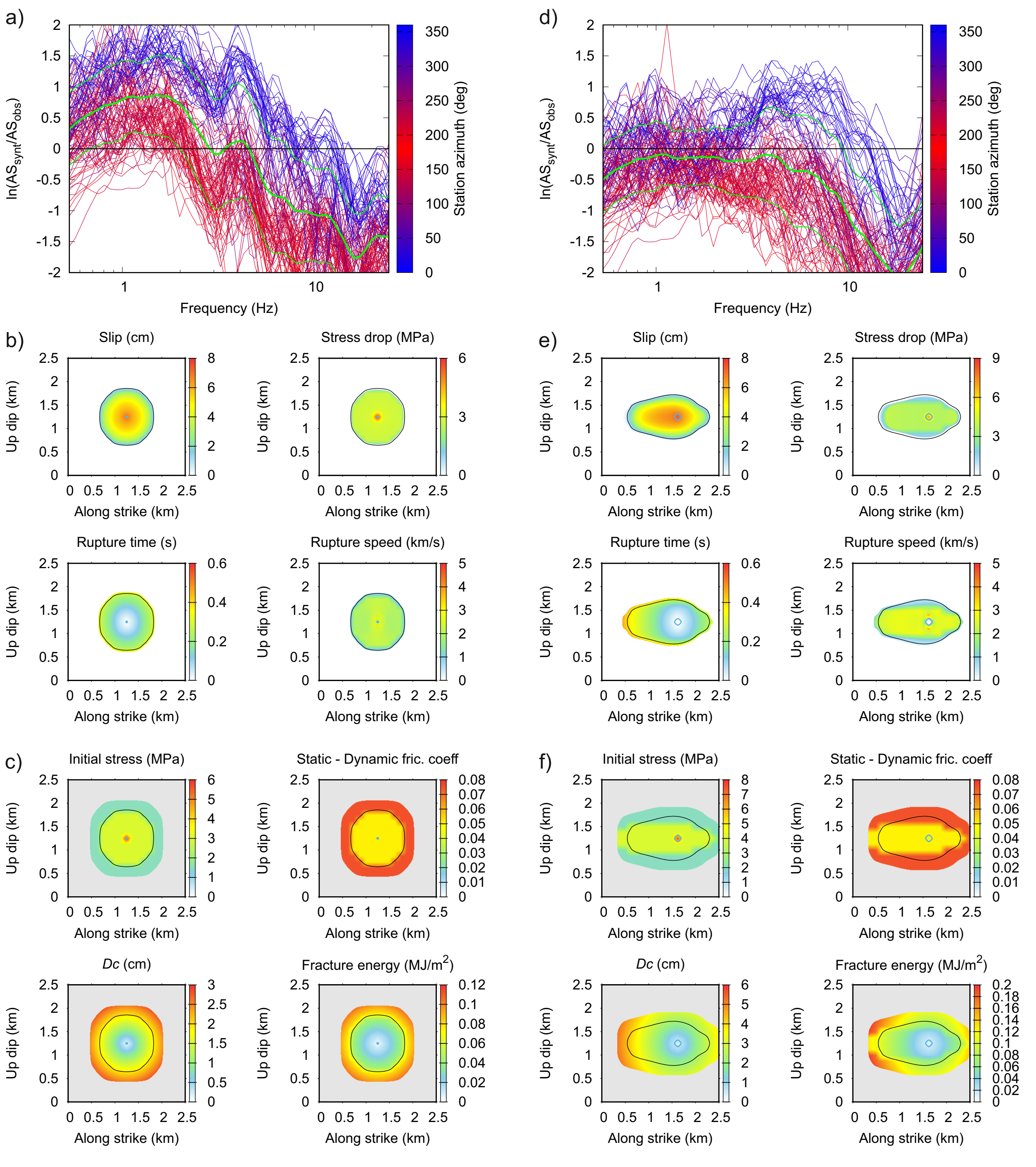

Figure S2: Illustration of the poor fit for the directive 2016-10-30 Mw4.2 event when using (a-c) a smooth symmetrical and (d-f) a smooth asymmetrical rupture model. (a,d) Modeling bias for all receivers as a function of frequency, color-coded by station azimuth. Thick and thin green lines represent the mean and standard deviations, respectively. Note that the model fails to reproduce the azimuthal dependency and decay of the spectra. (b,e) Kinematic and (c,f) dynamic rupture parameters of the respective rupture models.

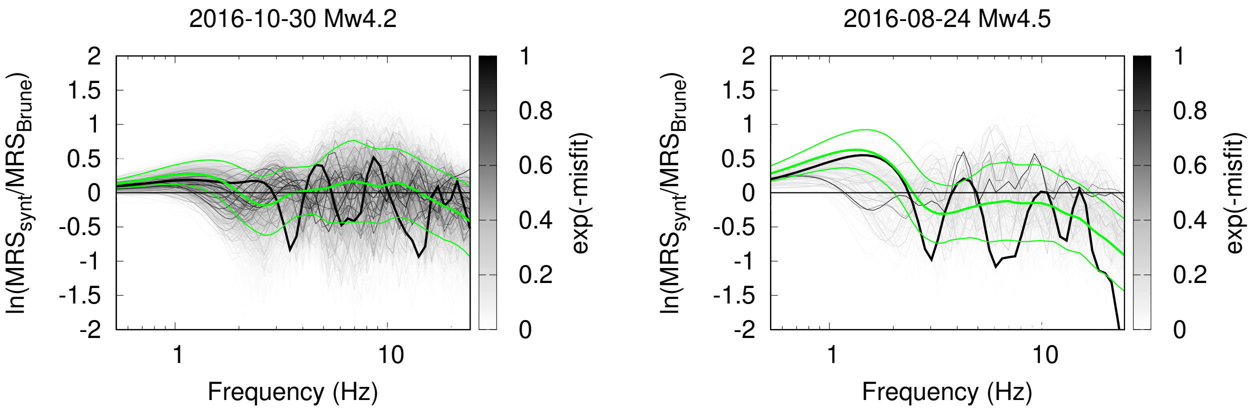

Figure S3: Results of the L2 optimization of a standard omega-squared model (Eq. 1) to fit the moment rate spectra (MRS) of each model in the inversion ensemble shown in terms of spectral bias (gray-shaded lines) for the (left) directive Mw4.2 event, and (right) nondirective Mw4.5 event. Thick and thin green lines show the average and one standard deviation spectral bias over the ensemble models, respectively. Thick black line depicts the bias of the maximum a-posteriori model. While the omega-squared model is a suitable approximation for the directive Mw4.2 event, the spectra of the non-directive Mw4.5 event decay faster at higher frequencies, resulting in generally lower misfit values.

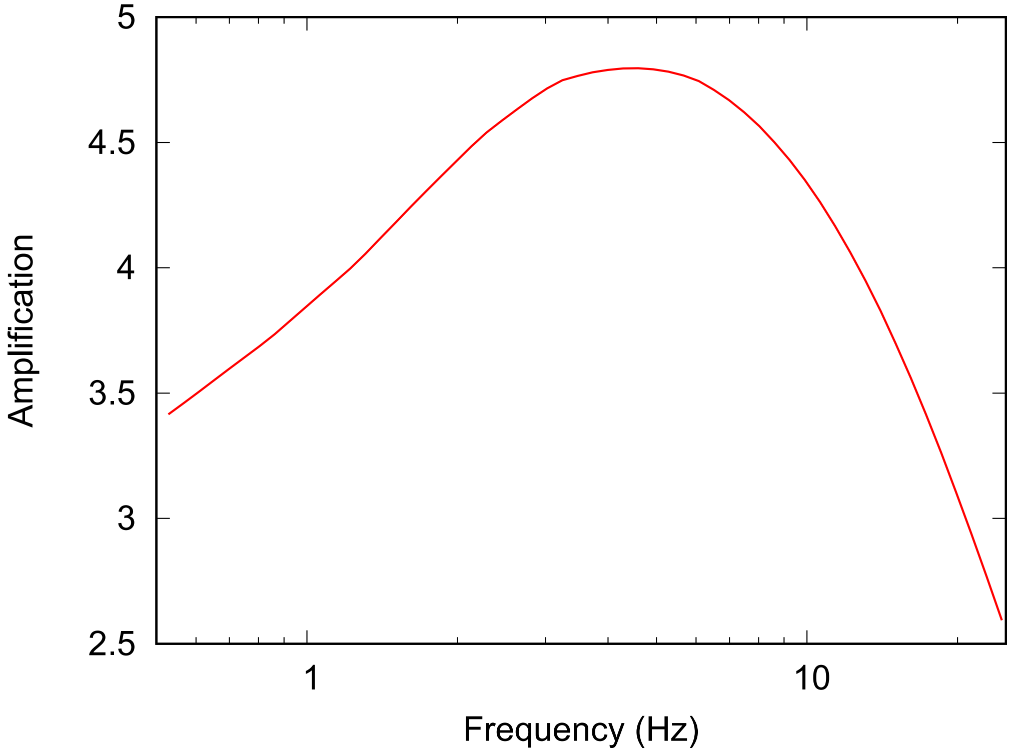

Figure S4: Source-to-surface amplification for a reference rock site, comprising of free surface amplification, generic rock site amplification by Boore and Joyner [1] , shallow attenuation modeled as kappa effect with $\kappa = 0.015\,\mathrm{s}$, and amplification due to the S-wave impedance (i.e., velocity times density) contrast between the surface and the source.

S1 Synthetic test

To explore the resolving power of the dynamic source inversion of the apparent spectra (AS), we design a synthetic test with a similar setting as the directive Mw4.2 Central Italy event (see the main text for more details).

The target model is based on a preliminary inversion result for the directive Mw4.2 event but with added patches of increased initial stress, friction coefficient drop $\Delta\mu$ (or strength) and D_c to enhance the model heterogeneity (see Figs. S5a and S6a for its kinematic and dynamic properties, respectively). The rupture of the target model starts in a small nucleation patch on the right side of the fault, propagates around a barrier created by a high $\Delta\mu$ (or strength) patch, and ruptures two stress patches on the left side, generating high slip asperities. In the end, the bypassed barrier of the high strength breaks (see the late rupture time in Fig. S5a). The rupture velocity is very heterogeneous with locally supershear values.

The resulting target AS for 117 stations (the same as in the real case), which serve as input data for the inversion, are shown as a function of frequency in Fig. S7a, color-coded by station azimuth. The spectra exhibit a clear directivity pattern with stronger radiation toward the south. We note that the target model's synthetic AS generally fit the empirical (obtained by Generalized Inversion Technique, GIT) AS of the directive event (spectral bias mean value is almost zero and the standard deviation is 0.48 in log). We do not add any additional noise to the target data.

The McMC inversion is run with the same settings as the real data inversion (see Tab. 1 of the main text), assuming the same a priori conditions, AS data error, and starting from the same set of initial models. To eliminate effects of different parameterization, we keep the same model parameter grid as in the target model. Because the inversion is performed using perfect (noiseless) data and employs the exact model parameterization and fault setup as the target model, it represents an idealized case, designed primarily to test the resolving power of the AS data. The synthetic inversion results in 1623 dynamic rupture models as our final model ensemble after discarding the first 20% samples of each chain as the burn-in phase.

To assess the role of prior constraints on the dynamic rupture inversion, we perform an additional McMC sampling with the same setting but without posterior constraints placed by the target AS. In this way, we sample only the prior distribution represented by the magnitude value with uncertainty, nucleation constraints, and the ranges of the dynamic parameters. We obtained 2000 dynamic models that sample the prior distribution, a similar number as for the posterior inversion. We admit that the representative prior sampling should be much more comprehensive than the posterior. However, for computational reasons, we stopped the sampler when the sampled marginal probability density function (PDF) on magnitude converged approximately to the prescribed prior PDF. Therefore, it can be considered as a rough estimate of the prior PDF.

S1.1 MAP models of the AS inversion

We compare the target model with the maximum a posteriori (MAP, with the lowest misfit) model and a secondary model resulting from a different McMC chain with the second lowest misfit of the ensemble. The mean spectral bias of the two models is -0.005 and -0.012 in log, and the root-mean-square errors are 0.314 and 0.319, respectively. The two models

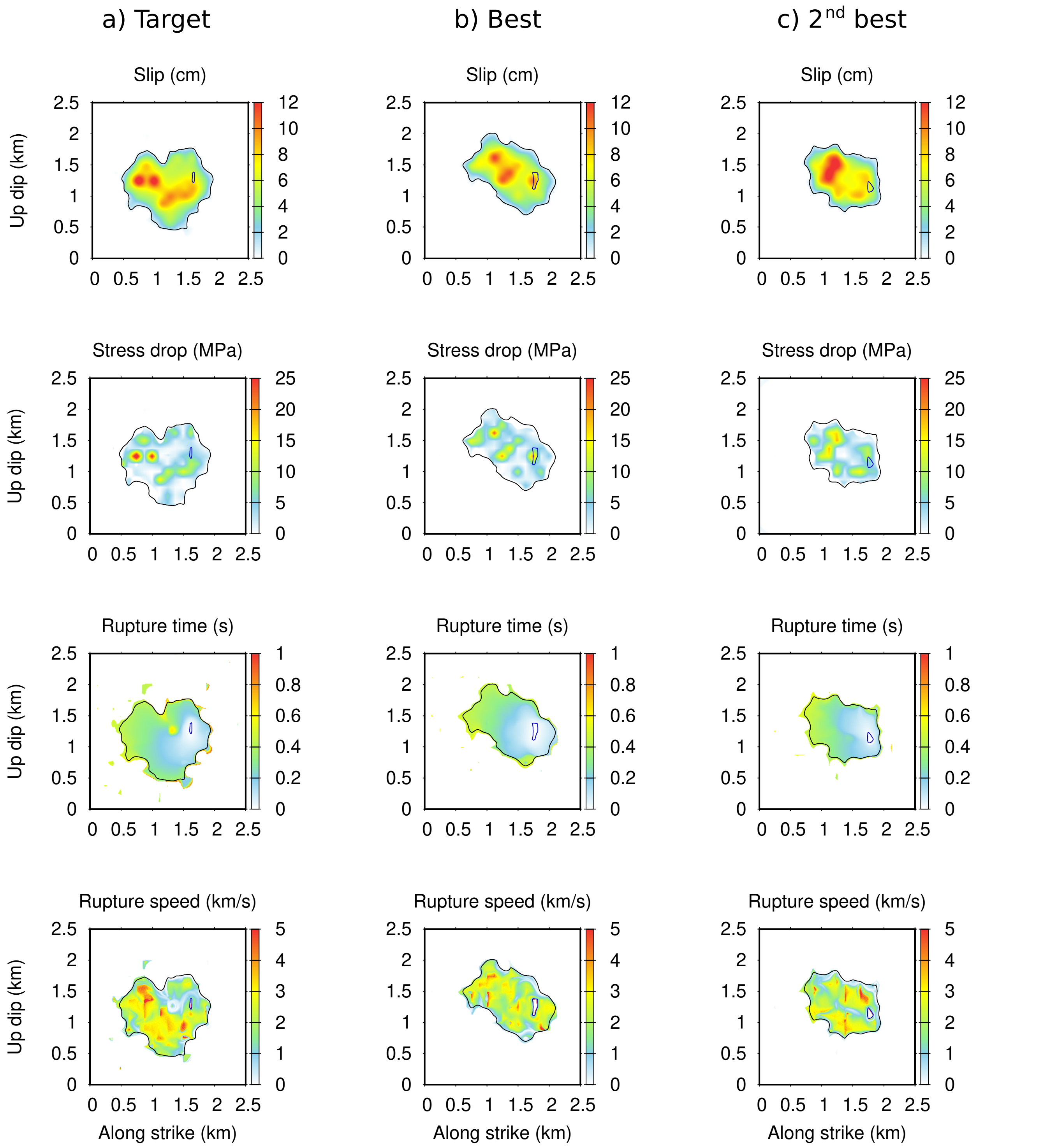

Figure S5: Kinematic rupture properties of the (a) target, (b) best (MAP), and c) 2nd best model (i.e., the best model from another Monte Carlo Markov chain); see panel legends. Blue contours outline the nucleation areas (i.e., the patches with negative strength excess), and black lines contour the slip distribution at 10% of its maximum.

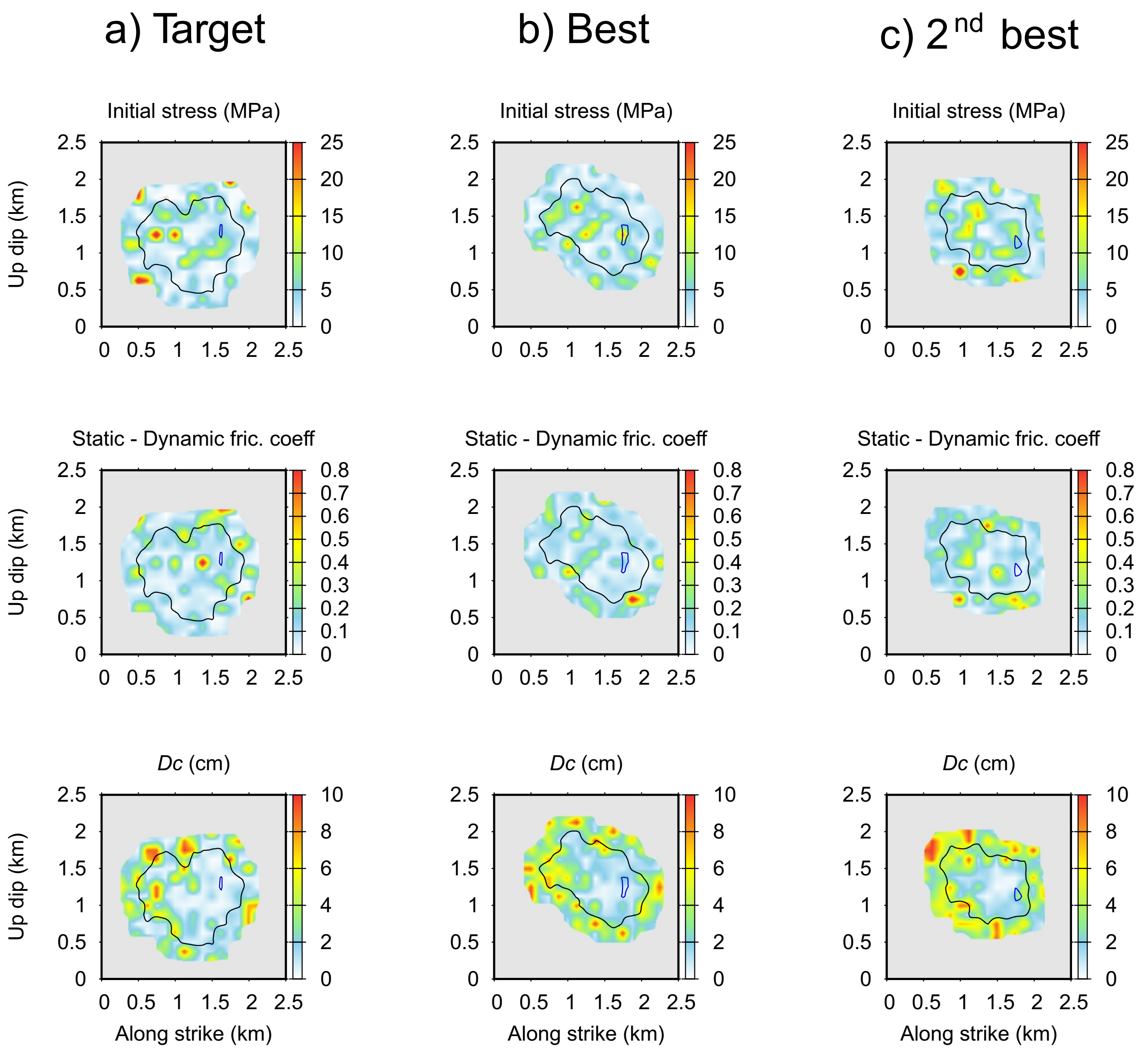

Figure S6: Dynamic rupture parameters of the (a) target, (b) best (MAP), and (c) 2nd best model (i.e.,the best model from another Monte Carlo Markov chain); see panel legends. Blue contours outline the nucleation areas (i.e., the patches with negative strength excess), and black lines contour the slip distribution at 10% of its maximum. Parts of the models that are unconstrained by the data are masked by grey.

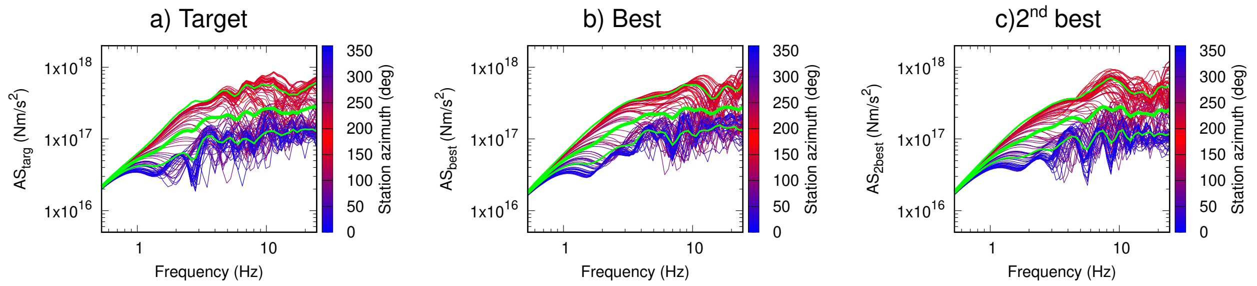

Figure S7: Synthetic apparent spectra (AS) of the (a) target, (b) best (MAP), and (c) 2nd best model (i.e., the best model from another Monte Carlo Markov chain), color-coded by station azimuth. Thick and thin green lines correspond to the mean and one standard deviation across stations, respectively.

with similar misfits are used to demonstrate the variability between the individual ensemble models.

The synthetic AS of the target model and the two inverted best models are shown in Fig. S7, demonstrating a southward directivity of all three models and a good agreement of the mean spectra and their variability over the stations. The resulting spectral biases of the best models are shown in Fig. S8 as a function of frequency for each station, and in map view for selected frequencies. The AS are very similar among stations at frequencies $< 1\,\mathrm{Hz}$, that is, below approximately the corner frequency, where the event radiates as a point source, and because the synthetics do not contain any additional errors or noise. The slight underestimation of the spectra at low frequencies results from the minor underestimation of the target moment in these two models (cf. also Fig. S11). At frequencies $> 1\,\mathrm{Hz}$, the variability of the AS and also of the spectral bias increases significantly. It resembles the real-data application (Fig. S1a left), likely due to assuming the same data error. The map view demonstrates that although the total misfit is similar for the two models, the spatial distribution of the spectral bias can vary: the best model misestimates the target AS in fault-parallel azimuth at 10 Hz, whereas the 2nd best model in the direction perpendicular to the fault. This suggests that the two best dynamic models differ significantly.

Fig. S5 shows the kinematic rupture properties of the target and the two inverted models. In general, the inverted models reproduce the directive rupture propagation from the nucleation, only slightly underestimating the duration and the rupture extent in the down-dip direction, resulting in a smaller seismic moment. The models show heterogeneous rupture characteristics demonstrated by the distribution of rupture velocity, slip, and stress drop. Nevertheless, the positions of the heterogeneities on the fault are not correlated with the target model. For example, patches with high slip values ($> 10\,\mathrm{cm}$) corresponding to high stress drop patches ($> 20\,\mathrm{MPa}$) are present in the inverted models but placed in different positions for both the inverted models and the target model.

The distributions of the dynamic rupture parameters of the target and the two best models are shown in Fig. S6. The resulting initial stress (with patches corresponding to the stress drop patches) shows a level of complexity comparable to that of the target model. Inverted $\Delta\mu$ and D_c are also heterogeneous along the fault. We note that both inverted models fail to recover the delayed rupture patch due to the high $\Delta\mu$ region of the target

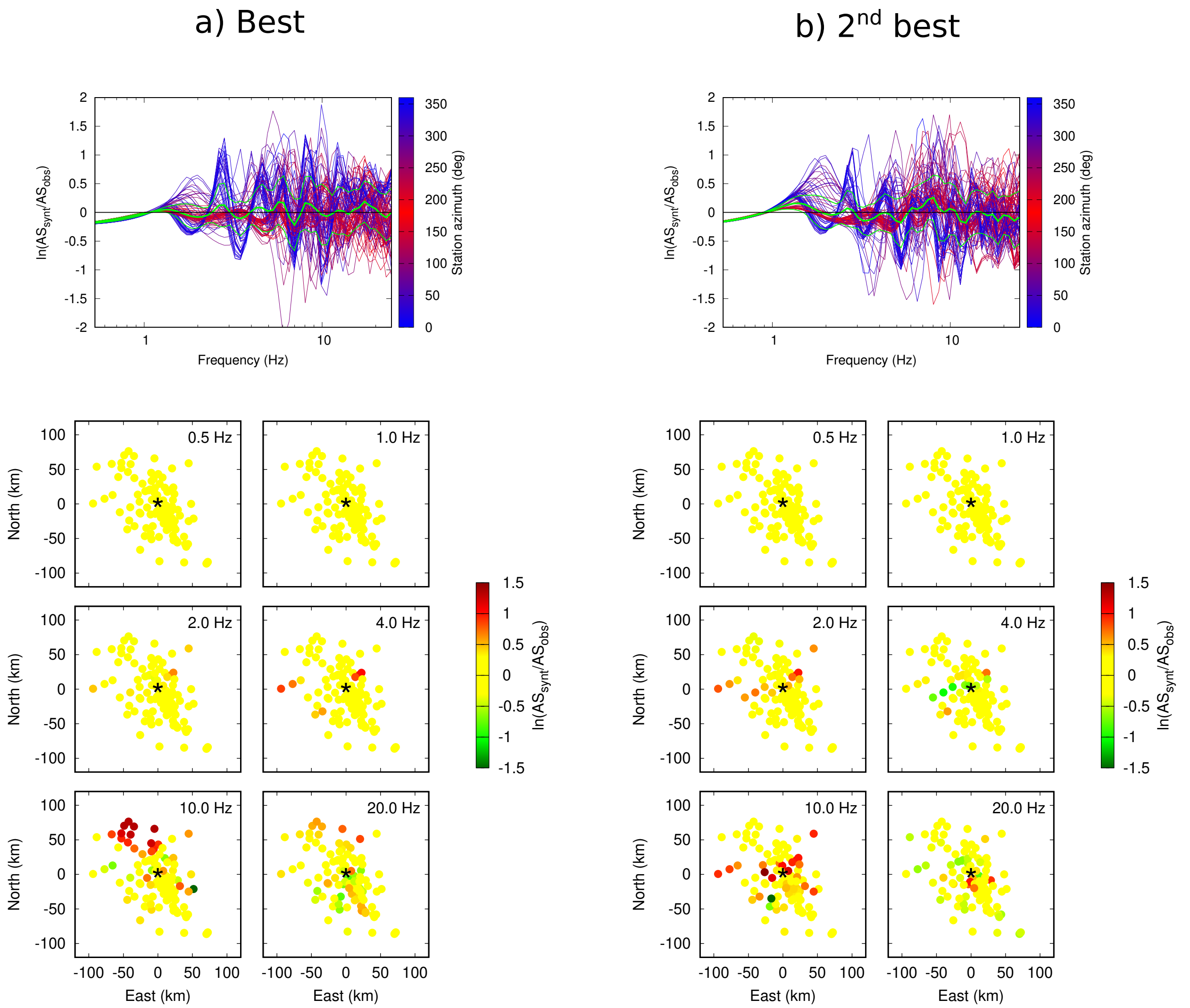

Figure S8: Spectral bias of (a) the best (MAP) and (b) the 2nd best model. Top panels: Spectral bias as a function of frequency across all stations, color-coded by the station azimuth. Thick and thin green lines represent the average and standard deviations, respectively. Bottom panels: Map view of the spectral bias at six selected frequencies (see legends). Black stars represent the epicenters.

model. This specific rupture patch was not recovered due to the limited McMC sampling, but since the spectral bias above the corner frequency is close to zero (Fig. S8), it is probably not necessary to fit the AS data in this frequency range. The analysis of the two best models suggests that the inversion results based on the AS cannot recover the authentic details of the target model. Instead, the inversion successfully reconstructs the heterogeneity of the target model, prompting the question whether it can resolve the event's mean characteristics.

S1.2 Ensemble properties of the AS inversion

Figs. S9a,b compare the moment rates (MRs) of all ensemble models and their second derivative (moment jerk) amplitude spectra with those of the target model. The models with the high PDF value (shown in darker color) are centered around the target model, but the whole ensemble shows very large variability. This results from (i) employing only the amplitude information of the spectra in the inversion, and (ii) assuming relatively large data error (4 in natural logarithm, corresponding to a factor of ∼50; see also Text S2).

Fig. S9b additionally shows the target AS averaged over stations and its variability (since it is standardly used for the MR estimate). Similarly to the real directive event, the station-averaged AS overestimates the moment-jerk spectra (Fig. 4a of the main text) and indicates that the moment-jerk spectrum lies closer to the lower bound of the AS variability. Nevertheless, the synthetic test confirms that this discrepancy does not negatively affect the dynamic rupture inversion of AS as the individual moment jerk spectra converge to the target spectrum.

Fig. S9c shows the average slip distribution over the whole ensemble and its contours compared to the target slip contour. Most of the slip of all the inverted models occurs after the nucleation maintaining the directive pattern, and the ensemble seems to underestimate slightly the down-dip rupture extent.

The ensemble-averaged distributions of dynamic and kinematic parameters along the fault are shown in Fig. S10. Compared to the individual models shown in Figs. S6 and S5, the averages do not exhibit prominent heterogeneities in any of the parameters presented. The prestress and friction drop are almost homogeneous, except for the increased prestress in the nucleation region (see also the stress drop distribution). The ensemble-averaged D_c shows an increase with distance from the nucleation, similar to that prescribed in the initial models to ensure constant rupture propagation from a small nucleation area. The smoothness of the averaged models further confirms that local heterogeneities in the individual ensemble models are located randomly and, except for the nucleation region, are uncorrelated across the ensemble–consistent with the real-data inversion, see the main text. We recall that the average smooth model cannot reasonably fit the AS of the target model as demonstrated in Fig. S2.

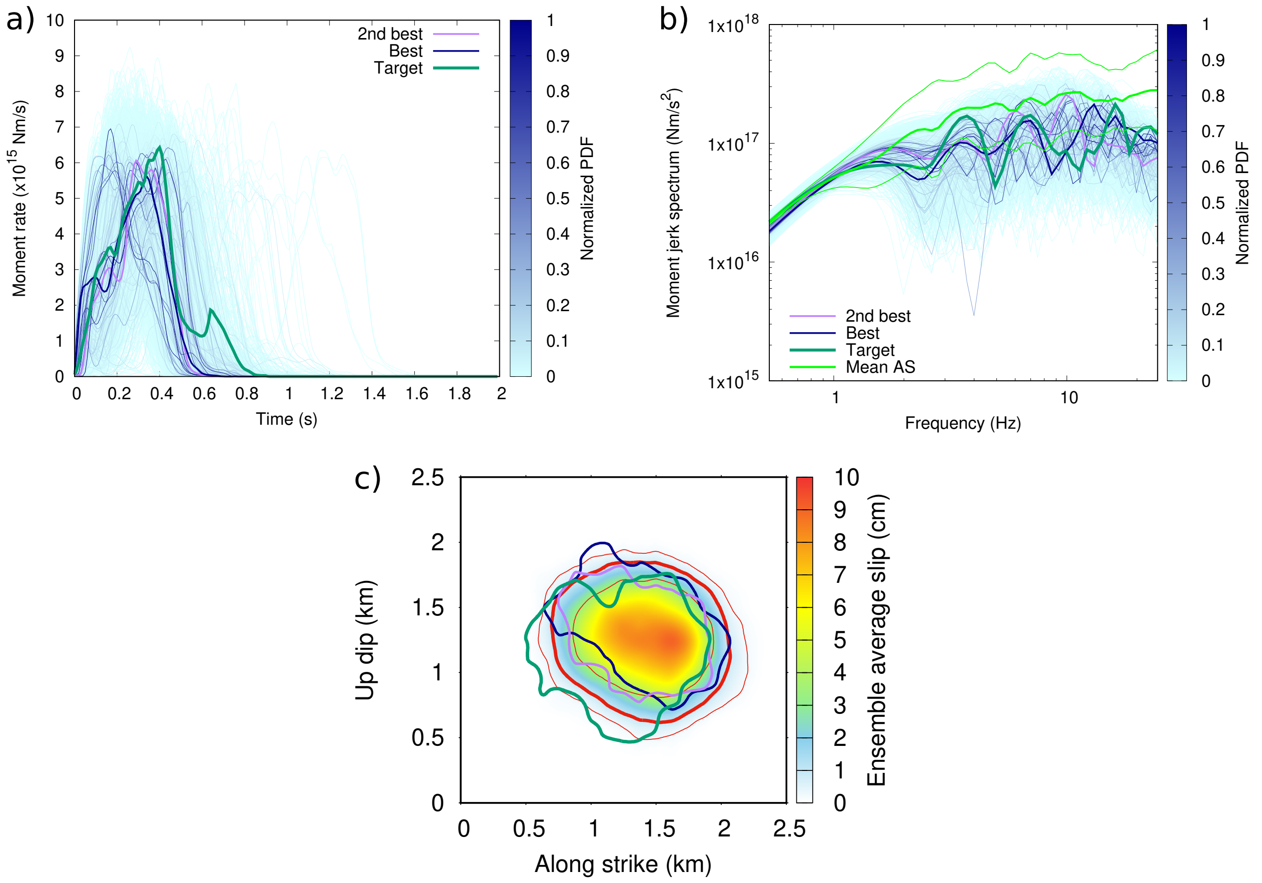

Figure S9: (a) Moment rates (MRs) of the ensemble models, color-coded by their normalized posterior probability density function (PDF) values. The two best models are highlighted by thick dark-blue and purple lines. The MR of the target model is shown by thick green line. (b) Smoothed amplitude spectra of the second time-derivative of the MR (moment jerk) of the ensemble models, color-coded by their normalized posterior PDF values. The two best models are shown by thick dark-blue and purple lines, and the target model is shown by darker green line. Light-green lines show the mean and standard deviation of the target model's apparent spectra (AS) across stations. (c) Ensemble-averaged slip distribution shown in color, with contours outlining the average slip (thick red line) and its standard deviation (thin red lines), defined at 10% of the maximum slip. The slip contours of the two best models are shown by dark-blue and purple lines, and the contour of the target model is shown in green.

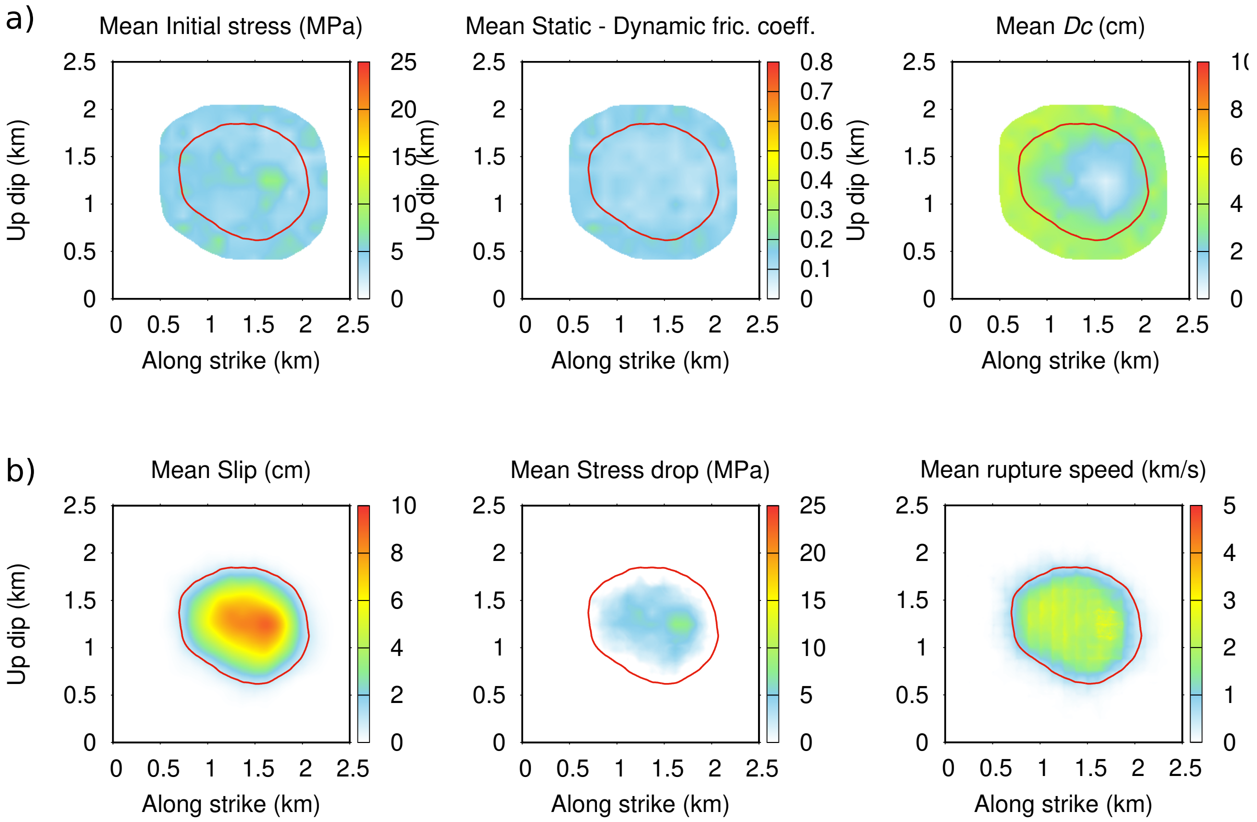

Figure S10: Ensemble averages of (a) the dynamic rupture parameters and (b) kinematic rupture properties; see panel legends. Parts of the dynamic model unconstrained by the data are not shown. The red contours outline the average slip distribution at 10% of its maximum.

S1.3 Comparison of prior and posterior marginal PDFs of average rupture parameters

Here, we examine the average source parameters of the inferred posterior PDF samples. We compare them with the target values and properties of the sampled prior PDF in Fig. S11 to inspect which parameters are resolvable by AS data and which are determined mainly by the prior constraints (i.e., magnitude constraint, parameter ranges, restricted nucleation). Tab. S1 lists log-normal mean and standard deviations for all parameters for quantitative comparison.

Fig. S11 demonstrates that the marginal posterior PDFs of all parameters estimated by the AS inversion cover the target model values. Moreover, in most cases, the target value corresponds to the PDF maximum and also to the values of the best models. A slight underestimation for the moment and duration is observed, likely due to a negative effect of the prior condition with the prescribed lower moment value. Most of the other parameters (mean slip, stress drop, and energy estimates) have narrower posterior PDFs than the prior PDF and are located at the tails of the prior PDF. It suggests that these parameters are estimated with a low impact of the prior constraints. The marginal posterior PDF of the rupture radius lies at the very upper tail of the prior distribution, yet with as large variability as the prior (cf. also Tab. S1). This parameter is clearly guided by the AS towards the target values despite the rather large uncertainty. Indeed, the unconstrained sampling tends towards smaller ruptures with higher slip.

The posterior PDF maximum of the radiation efficiency correlates with the target value, but also with the maximum of its prior. Nevertheless, the posterior PDF has a narrower distribution than the prior. The least resolved parameters, according to our test, are the rupture velocity and the rupture duration, showing a small difference between the prior and posterior PDFs. We note that both the prior and the posterior are implicitly constrained also by the rupture dynamics so that the slip rates satisfy the elastodynamic equation and the assumed friction law. The limited range of mean rupture velocity over the prior samples is therefore primarily governed by the dynamic model with heterogeneous parameters. Alternatively, it may result from the limited sampling of the dynamic parameter space, in particular D_c, and initiating the sampler using models with prescribed D_c increase that does not contradict the mean rupture behavior (e.g., see also the average model in Fig. S10).

We conclude that, according to our synthetic test, the dynamic inversion of the AS can reliably estimate most of the main parameters of the earthquake source. In particular, we find a good sensitivity for the mean stress drop, and fracture and radiated energy estimates. This implies that, e.g., the similarity of the mean stress drops of the two real events (see main text) can reflect the constant stress drop scaling in the tectonic setting in Central Italy rather than the AS's insensitivity to this parameter.

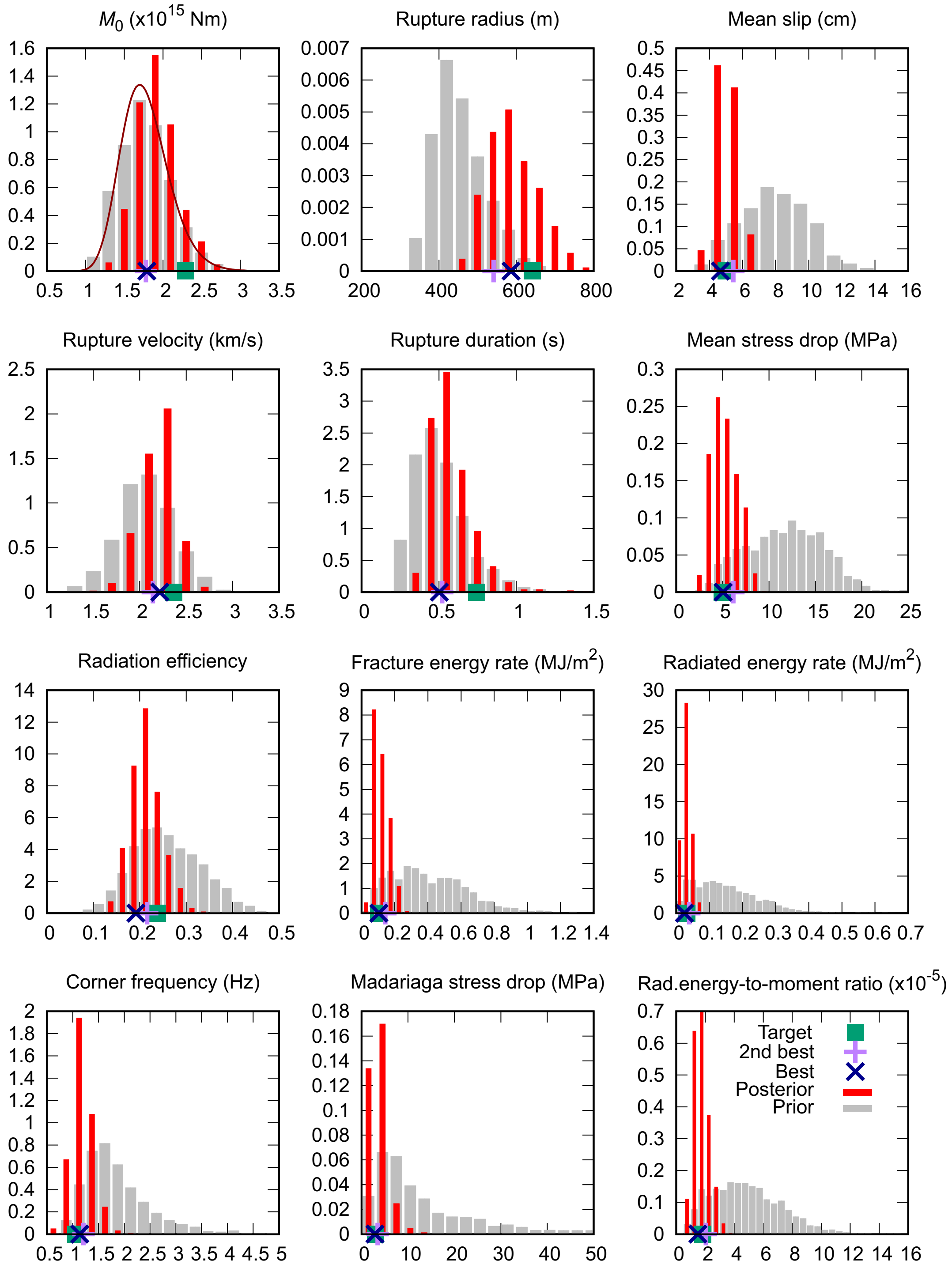

Figure S11: Histograms of mean parameters (see panel legends) of the model ensemble, normalized to unit integral, representing the marginal posterior distributions in red. Gray histograms show the normalized marginal distribution of the prior (see details in the text). Green squares mark the values of the target model. Dark-blue crosses and purple pluses show the best (MAP) and 2nd best models, respectively. Note that the prior distribution of the Madariaga stress drop is very long-tailed (extending up to 400 MPa); for clarity, we limited the value ranges in the plot.

Table S1: Mean and standard deviation derived from log-normal fits to the marginal probability density functions (PDFs) of the parameters shown in Fig. S11, both prior and posterior. The last column presents the corresponding values of the target model.

Parameter	Prior mean ± stdev	Posterior mean ± stdev	Target
M_0 (x10^{15} Nm)	1.72 ± 0.34	1.88 ± 0.26	2.2912
Rupture radius (m)	437.27 ± 61.41	578.98 ± 67.31	641.0450
Mean slip (cm)	7.77 ± 2.27	4.93 ± 0.73	5.0072
Mean rupture velocity (km s^{-1})	2.06 ± 0.29	2.21 ± 0.18	2.3672
Duration (s)	0.45 ± 0.16	0.54 ± 0.11	0.7440
Mean stress drop (MPa)	11.19 ± 5.03	4.87 ± 1.55	4.9671
Radiation efficiency	0.25 ± 0.08	0.21 ± 0.03	0.2381
Fracture energy rate (MJ m^{-2})	0.31 ± 0.28	0.10 ± 0.05	0.1035
Radiated energy rate (MJ m^{-2})	0.09 ± 0.13	0.03 ± 0.01	0.0323
Corner frequency (Hz)	1.61 ± 0.49	1.15 ± 0.20	1.0620
Madariaga stress drop (MPa)	6.40 ± 6.61	3.05 ± 1.46	3.0238
Radiated energy/M_0 (x10^{-5})	3.97 ± 2.85	1.58 ± 0.53	1.8220

S2 Note on data uncertainty

The misfit considered in the likelihood function is assumed to be the L2 norm between the natural logarithms of empirical and synthetic apparent spectra (AS) normalized by the square of data error. We assume that the data standard deviation is constant and equal to 4. Despite this value was found rather empirically, we provide below a rationale justifying this choice. Theoretically, if the assumed data error is correct and the data are independent, the normalized L2 norm should equal the number of data (56 frequencies times approximately 100 stations, i.e., 5,600). As shown in the Results section, our normalized L2 norm reaches around 50, i.e., approximately 100 times lower. This can be interpreted either as the data error being a conservative estimate ($\sim$10 times larger than the true error), or as an indication that the data are not strictly independent. Indeed, it is important to note that many stations are close to each other (Fig. 1a), and that the spectra are smoothed, making the 100-fold data reduction reasonable. We also point out that while the data error value dictates the overall uncertainty of the model, the relative uncertainties among the individual parameters and their potential trade-offs remain unaffected.

Alternatively, the estimate of the data variance can be guided by the standard deviation of the AS at low frequencies (i.e., below the corner frequency) where the AS should collapse to a common (station-independent) value. Fig. S12 shows the standard deviation of the AS for each event as a function of frequency. The value below 1 Hz oscillates between 0.4 and 0.6 in logarithm, which is again 10 times lower than our conservatively assumed error in the Bayesian inversion.

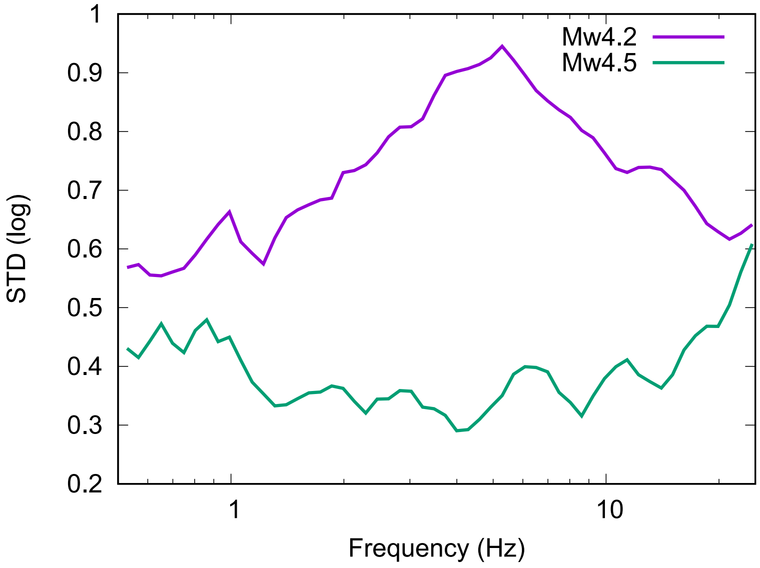

Figure S12: Standard deviation of the apparent spectra (AS) over all stations (STD) as a function of frequency for the two events (see legend). Below the corner frequencies (∼1 Hz), the curves can be used to assess the standard deviation of the AS data because they should theoretically approach zero.

S3 Limitations of the methodology

The proposed method is based on the inversion of apparent source spectra corrected for any site or path amplification effect, which is challenging to extract from real data. When using the Generalized Inversion Technique (GIT), reference sites for GIT must be selected carefully, and any residual amplification of the reference sites must be carefully considered. It is possible that the dynamic inversion results are vulnerable to the residual complexities of site and path effects that are not considered in the GIT method. Indeed, both site and path effects can be azimuthally or source-depth dependent, stemming from 3D wave propagation effects, which would be artificially shifted by the GIT method to the apparent source spectra. Nevertheless, when using many stations (>100 in our case), the residual effects should manifest as unexplained random posterior data errors, perhaps only weakly affecting the inverted source models.

The inversion result will also depend on the details of the removal of the residual site amplification (the source-to-surface amplification of the reference rock site, including κ value) and the (average) radiation pattern. We point out that such a choice needs to be made in all empirical methods based on spectral fitting and is thus common to all methods aiming to infer rupture properties of earthquakes. For example, the choice of κ value was selected to be consistent with rock site conditions in Central Italy [2, 3]. Nevertheless, the specific choice introduces a systematic effect to the apparent spectra, e.g., a smaller value (0.01 s) results in smaller spectral decay at high frequencies, which might be translated to enhanced small-scale dynamic (and thus kinematic) parameters. Further research might help to improve the characterization of the residual spectral amplification and thus improve the source parameter estimates.

We also point out that we use an idealistic planar fault in the present application. Since the true rupture is affected by various physical (here unmodeled) properties, such as fault nonplanarity, off-fault damage, inhomogeneous fault rheology, etc., the inferred dynamic parameters are to be considered effective. Nevertheless, the direct association of our results with such complexities is beyond the scope of the present paper, but it can be an aim of further research.

Yet another simplification comes from neglecting the centroid moment tensor and location uncertainties in the dynamic inversion. The only exception is the uncertainty in Mw, which serves as a prior, depicted in the histogram of M_0 in Fig. 6. Note that a Gaussian PDF is assumed in Mw, which corresponds to a log-normal PDF in M_0 (see Fig. 6). Since the dynamic inversion is formulated in the Bayesian framework, the centroid and location uncertainties could be propagated to the uncertainty of the source properties. This can be included in the further development of the proposed dynamic inversion.

S4 Performance of smooth models with strong barrier

The smooth models shown in Fig. S2 do not fit the high-frequency content of the observed source spectra. The models arrest gradually due to the absent or weak barrier, and thus do not generate enough high-frequency radiation. Indeed, smooth rupture models encountering instantaneous stopping phases due to the strong barrier are able to generate ω-squared spectra, as found by [4] and extensively studied by [5]. Here we inspect the performance of such models to fit the real spectra.

To keep the problem simple, we target the non-directive Mw 4.5 event. In this case, the geometry of the rupture is circular with the hypocenter located in the center of the fault, i.e., we can fix the nucleation position unlike in the case of the Mw 4.2 directive model. Assuming a fixed moment, we perform a simple parametric study for two independent parameters - stress drop and rupture velocity. The resulting models are assessed based on their misfit with the observed apparent spectra (AS). We keep the same model parameterization as in the case of heterogeneous models, i.e, 100 m. We emphasize that we present only a limited number of simulations, and thus, the results presented in this section are not to be confronted with the results of the Bayesian dynamic inversion presented in the main text.

The stress drop value is controlled by the initial stress value within the circular rupture patch. Both the initial stress and strength are prescribed to be homogeneous within the circle. Outside the circle, the initial stress drops to zero and strength increases 15 times, generating a strong barrier. The nucleation is prescribed in the center of the circle by increasing the initial stress above the strength in a small patch. We tested 3 initial stress values: 2 MPa, 4 MPa, and 8 MPa. We note that the resulting mean stress drop $\Delta\sigma$ differs from the initial stress values in the homogeneous model. Since the stress drop controls the size of the circular rupture, we use the scaling relation between M_0 and r to set the radius of the circle of the homogeneous model.

The second parameter, the rupture velocity, can be controlled by D_c of the linear slip-weakening friction law. In our approach, it is determined by the D_c-rate, which describes the assumed linear increase of D_c from the nucleation according to Eq. (17) in the main text. We assumed several values of D_c-rate that result in mean rupture velocity ranging between 0.4 and 1.0 of β.

This setting provides models with almost constant stress drop and rupture velocity along the fault. However, to rigorously represent the results, we evaluated the mean stress drop and the mean rupture velocity in the area with non-zero slip. We note that as these parameters vary slightly from the assumed relations, there is also a small variation in the resulting M_0.

For the inspected barrier dynamic models, we compare the synthetic apparent spectra with the observed ones, see Fig. S13. In this set of models, the best model with misfit ~ 52 could have been accepted in the ensemble of the Bayesian inversion, if it had been present in the McMC sampling. The kinematic and dynamic parameters and the spectral bias of the best model are shown in Fig. S14

Based on this simple parametric study, we can infer some general characteristics of the barrier model necessary to fit the observed apparent spectra. The best model has a stress drop of ~ 4.2 MPa; models with stress drop values of ~ 2 and ~ 8 MPa have a higher misfit (> 70). The preferred stress drop value lies within the marginal posterior distribution from the Bayesian inversion (cf. Fig. 6).

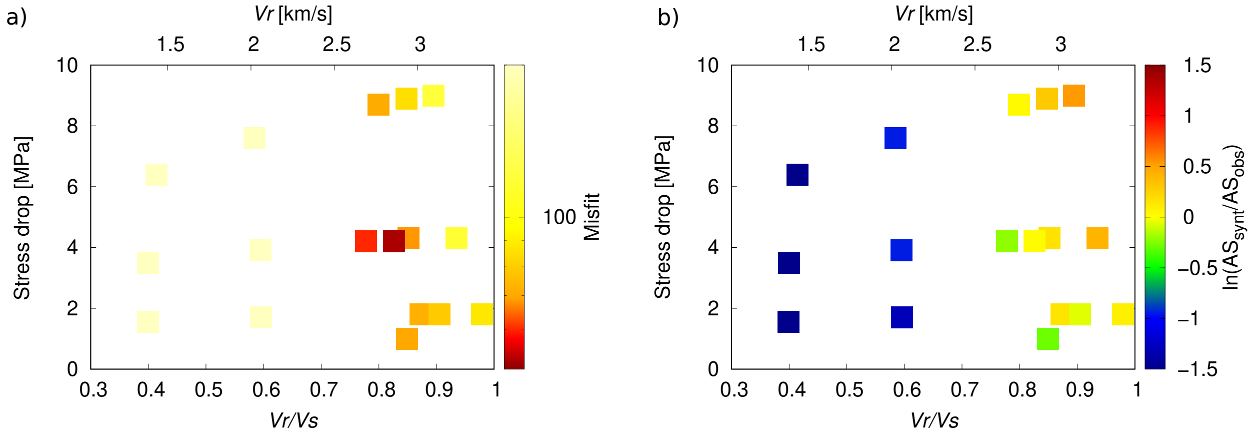

Figure S13: Results of the parametric study of the smooth models with barrier for the non-directive Mw 4.5 event. The individual models are represented by their stress drop and rupture velocity v_r (or alternatively as ratio between rupture velocity and S-wave velocity v_r/v_s). The individual models are color-coded by a) the misfit, and b) the mean spectral bias. The parametric study shows the preference of higher rupture velocity to reproduce the observed source spectra. The best model is characterized by the stress drop of 4.2 MPa and rupture velocity of 2.9 km s^{-1}.

In contrast to the heterogeneous model, the barrier model requires a higher rupture speed (2.9 km s^{-1} or $v_r/\beta \sim 0.8$). The rupture is required to hit the barrier with enough energy to generate high-frequency radiation comparable with the observations. For slower ruptures, the synthetic spectra are weaker than the observed ones, resulting in negative spectral bias, whereas faster ruptures result in too strong spectral radiation and thus positive spectral bias. In addition, there is a small negative trade-off between the rupture velocity and the stress drop. We remind that in the heterogeneous models found by the Bayesian inversion, the high mean rupture speed was not necessary, as rupture includes small-scale accelerations and decelerations throughout the whole evolution and thus allows smaller values of mean rupture velocities (< 2.7 km s^{-1}, see Fig. 6).

We note that the barrier and heterogeneous models differ in radiation efficiency (~ 0.2 and ~ 0.3 for the heterogeneous and barrier models, respectively). Unlike for the rupture velocity, this difference is not as distinctive because the value of 0.3 lies in the upper uncertainty bound of the marginal posterior PDF from the Bayesian inversion.

Since the average rupture velocity cannot be inferred directly from amplitude spectral data, the barrier model can be considered as a complementary approach to heterogeneous-down-to-small-scale parameterization. However, numerous studies in various fields suggest that faults exhibit heterogeneity over a wide range of scales, e.g., laboratory experiments [6, 7, 8], field observations of natural faults [9, 10, 11], and strong motion simulations [12, 13, 14, 15, 16, 17]. Properly addressing the comparison between the heterogeneous and smooth barrier models requires an in-depth study, including an exhaustive parametric study of the barrier model and additional data sensitive to rupture velocity, so we keep it for future work.

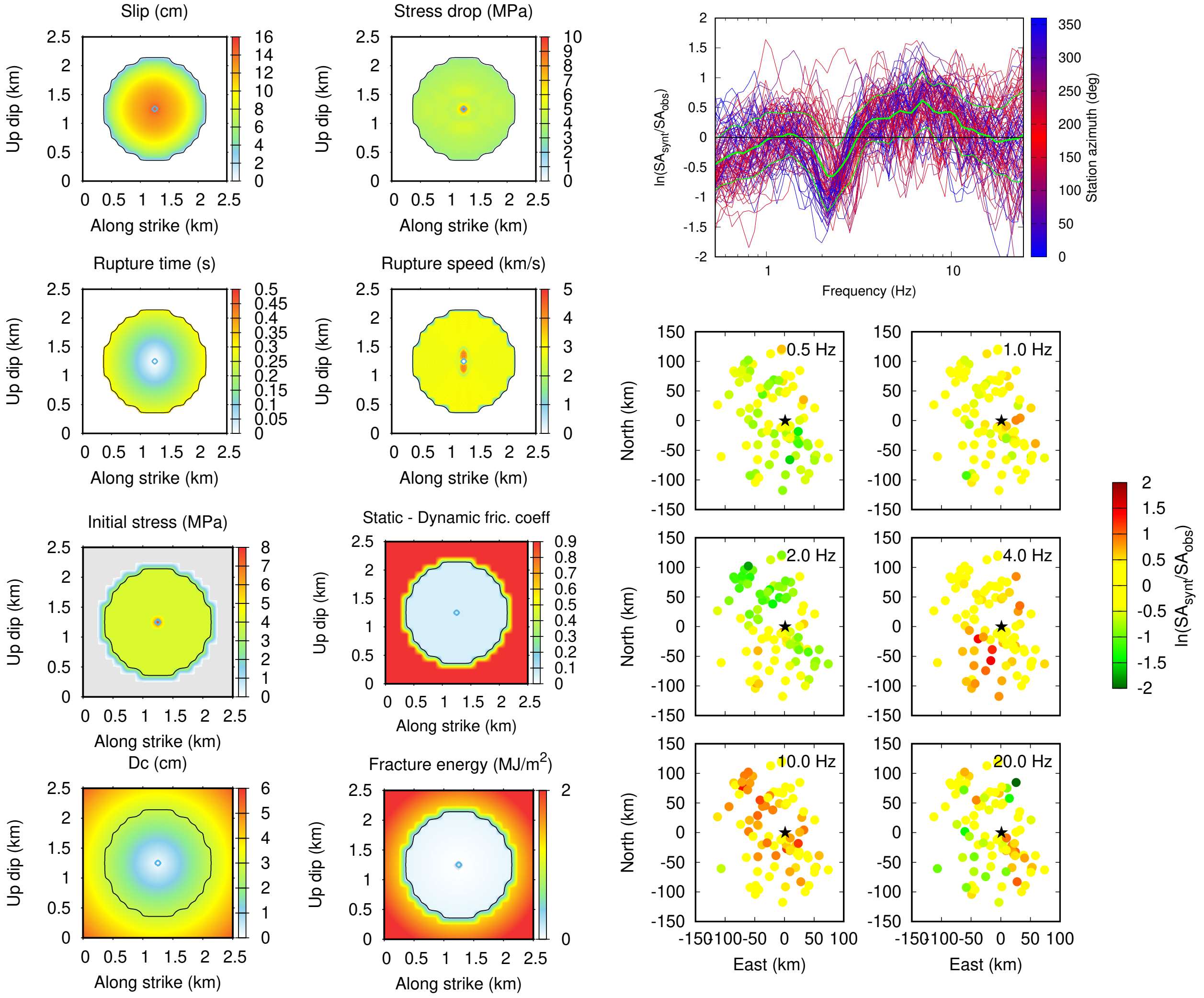

Figure S14: Properties of the best smooth model with a strong barrier from the parametric study for the non-directive Mw 4.5 event: a) kinematic rupture properties; see panel legends; b) dynamic rupture parameters; see panel legends. In a) and b), blue contours outline the nucleation, black lines contour the slip distribution at 10% of its maximum. c) Spectral bias as a function of frequency across all stations, color-coded by the station azimuth. Thick and thin green lines represent the average and standard deviations, respectively. d) Map view of the spectral bias values at each station for six selected frequencies (see legends). Black star represents the epicenter.

References

[1] Boore, D. M. & Joyner, W. B. Site amplifications for generic rock sites. *Bulletin of the Seismological Society of America* **87**, 327–341 (1997).

[2] Lanzano, G., Felicetta, C., Pacor, F., Spallarossa, D. & Traversa, P. Generic-To-Reference Rock Scaling Factors for Seismic Ground Motion in Italy. *Bulletin of the Seismological Society of America* **112**, 1583–1606 (2022).

[3] Morasca, P. *et al.* Empirical correlations between an FAS non-ergodic ground motion model and a GIT derived model for Central Italy. *Geophysical Journal International* **233**, 51–68 (2023).

[4] Madariaga, R. Dynamics of an expanding circular fault. *Bulletin of the Seismological Society of America* **66**, 639–666 (1976).

[5] Kaneko, Y. & Shearer, P. M. Variability of seismic source spectra, estimated stress drop, and radiated energy, derived from cohesive-zone models of symmetrical and asymmetrical circular and elliptical ruptures. *Journal of Geophysical Research: Solid Earth* **120**, 1053–1079 (2015).

[6] Schmittbuhl, J., Schmitt, F. & Scholz, C. Scaling invariance of crack surfaces. *Journal of Geophysical Research: Solid Earth* **100**, 5953–5973 (1995).

[7] Ohnaka, M. A constitutive scaling law and a unified comprehension for frictional slip failure, shear fracture of intact rock, and earthquake rupture. *Journal of Geophysical Research: Solid Earth* **108** (2003).

[8] Scholz, C. H. *The mechanics of Earthquakes and Faulting* (Cambridge University Press, 2002).

[9] Bistacchi, A. *et al.* Fault Roughness at Seismogenic Depths from LIDAR and Photogrammetric Analysis. *Pure and Applied Geophysics* **168**, 2345–2363 (2011).

[10] Candela, T. *et al.* Roughness of fault surfaces over nine decades of length scales. *Journal of Geophysical Research: Solid Earth* **117** (2012).

[11] Renard, F. & Candela, T. *Scaling of Fault Roughness and Implications for Earthquake Mechanics*, chap. 10, 195–215 (American Geophysical Union (AGU), 2017).

[12] Shi, Z. & Day, S. M. Rupture dynamics and ground motion from 3-D rough-fault simulations. *Journal of Geophysical Research: Solid Earth* **118**, 1122–1141 (2013).

[13] Ripperger, J., Mai, P. M. & Ampuero, J.-P. Variability of Near-Field Ground Motion from Dynamic Earthquake Rupture Simulations. *Bulletin of the Seismological Society of America* **98**, 1207–1228 (2008).

[14] Baumann, C. & Dalguer, L. A. Evaluating the Compatibility of Dynamic Rupture-Based Synthetic Ground Motion with Empirical Ground-Motion Prediction Equation. *Bulletin of the Seismological Society of America* **104**, 634–652 (2014).

[15] Withers, K. B., Olsen, K. B., Shi, Z. & Day, S. M. Validation of Deterministic Broadband Ground Motion and Variability from Dynamic Rupture Simulations of Buried Thrust Earthquakes. *Bulletin of the Seismological Society of America* **109**, 212–228 (2019).

[16] Taufiqurrahman, T., Gabriel, A.-A., Ulrich, T., Valentová, L. & Gallovič, F. Broadband Dynamic Rupture Modeling With Fractal Fault Roughness, Frictional Heterogeneity, Viscoelasticity and Topography: The 2016 Mw 6.2 Amatrice, Italy Earthquake. *Geophysical Research Letters* **49**, e2022GL098872 (2022).

[17] Gallovič, F. & Valentová, L. Broadband Strong Ground Motion Modeling Using Planar Dynamic Rupture With Fractal Parameters. *Journal of Geophysical Research: Solid Earth* **128**, e2023JB026506 (2023).
