# Supplementary material for: Physics-based broadband characterization of weak earthquakes
Source: Nat Commun. 2025 Dec 8;16:11300. doi: 10.1038/s41467-025-66461-w (PMC12722209; doi:10.1038/s41467-025-66461-w)
Supplement: Supplementary file 4 — Description of Additional Supplementary File [file 41467_2025_66461_MOESM4_ESM.pdf]

Description of Additional Supplementary Files

Supplementary Movie S1 :

Rupture propagation of the MAP model of the directive 2016-10-30 Mw4.2 earthquake (slowed down 10 times).

Supplementary Movie S2 :

Rupture propagation of the MAP model of the nondirective 2016-08-24 Mw4.5 earthquake (slowed down 10 times).
